# Semantic Integration of Multi-Modal Data and Derived Neuroimaging Results Using the Platform for Imaging in Precision Medicine (PRISM) in the Arkansas Imaging Enterprise System (ARIES)

**DOI:** 10.3389/frai.2021.649970

**Published:** 2022-02-10

**Authors:** Jonathan Bona, Aaron S. Kemp, Carli Cox, Tracy S. Nolan, Lakshmi Pillai, Aparna Das, James E. Galvin, Linda Larson-Prior, Tuhin Virmani, Fred Prior

**Affiliations:** ^1^Department of Biomedical Informatics, University of Arkansas for Medical Sciences (UAMS), Little Rock, AR, United States; ^2^Neurocognitive Dynamics Laboratory, Psychiatric Research Institute, University of Arkansas for Medical Sciences (UAMS), Little Rock, AR, United States; ^3^Department of Psychiatry, College of Medicine, University of Arkansas for Medical Sciences (UAMS), Little Rock, AR, United States; ^4^Department of Neurology, College of Medicine, University of Arkansas for Medical Sciences (UAMS), Little Rock, AR, United States; ^5^Department of Neurology, Comprehensive Center for Brain Health, University of Miami Miller School of Medicine, Miami, FL, United States; ^6^Department of Radiology, College of Medicine, University of Arkansas for Medical Sciences (UAMS), Little Rock, AR, United States

**Keywords:** imaging informatics, knowledge representation, ontologies (artificial intelligence), semantic web, neuroinformatics

## Abstract

Neuroimaging is among the most active research domains for the creation and management of open-access data repositories. Notably lacking from most data repositories are integrated capabilities for semantic representation. The Arkansas Imaging Enterprise System (ARIES) is a research data management system which features integrated capabilities to support semantic representations of multi-modal data from disparate sources (imaging, behavioral, or cognitive assessments), across common image-processing stages (preprocessing steps, segmentation schemes, analytic pipelines), as well as derived results (publishable findings). These unique capabilities ensure greater reproducibility of scientific findings across large-scale research projects. The current investigation was conducted with three collaborating teams who are using ARIES in a project focusing on neurodegeneration. Datasets included magnetic resonance imaging (MRI) data as well as non-imaging data obtained from a variety of assessments designed to measure neurocognitive functions (performance scores on neuropsychological tests). We integrate and manage these data with semantic representations based on axiomatically rich biomedical ontologies. These instantiate a knowledge graph that combines the data from the study cohorts into a shared semantic representation that explicitly accounts for relations among the entities that the data are about. This knowledge graph is stored in a triple-store database that supports reasoning over and querying these integrated data. Semantic integration of the non-imaging data using background information encoded in biomedical domain ontologies has served as a key feature-engineering step, allowing us to combine disparate data and apply analyses to explore associations, for instance, between hippocampal volumes and measures of cognitive functions derived from various assessment instruments.

## Introduction

Neuroimaging is among the most active research domains for the creation and management of open-access data repositories (Eickhoff et al., 2016). Structural and functional characteristics of the human brain can be measured using a range of neuroimaging techniques, such as magnetic resonance imaging (MRI), positron emission tomography (PET), magnetoencephalography (MEG), and electroencephalography (EEG). Many of the measures derived from these neuroimaging techniques have revealed characteristic differences among individuals who suffer from various brain disorders. Research investigations of brain disorders frequently employ neuroimaging in conjunction with established assessment instruments that have been venerated by years of clinical usage and/or validated as clinically meaningful measures of a given symptom, behavior, or functional domain of interest. Use of such condition-specific measures across studies poses unique challenges, particularly when mining neuroimaging data repositories that were originally collected for specific brain disorders, such as the Alzheimer's Disease Neuroimaging Initiative (ADNI) (Mueller et al., 2005) or the Progressive Parkinson's Markers Initiative (PPMI) (Marek et al., 2011). Identifying putative endophenotypic markers of impaired functional processes to distinguish various clinical conditions requires common semantic representations across both the neuroimaging data, as well as the diverse set of assessment instruments applied. Integrating shared semantic representations across the neuroimaging data, derived imaging measures, and associated non-imaging assessment data is therefore a critical step in seeking to apply reasoning and inferencing to detect either common or discriminative patterns of association across various brain disorders.

Drawing on our experience with open-access data publication and the software systems required to support such publication in The Cancer Imaging Archive (TCIA) (Clark et al., 2013), our research team is currently developing a streamlined containerized open-source software platform for the creation of imaging centric data repositories: PRISM (Platform for Imaging in Precision Medicine; prismtools.dev) (Sharma et al., 2020). One of the first applications of PRISM is the Arkansas Imaging Enterprise System (ARIES), which serves as a research data management system for the University of Arkansas for Medical Sciences (UAMS) and associated researchers. As the first instantiation of the PRISM infrastructure, ARIES also serves as a testbed to explore the practical utility and usability of the full set of capabilities that this new platform provides. The focus of this manuscript is the knowledge representation and reasoning approach used within ARIES to integrate semantic representations of multi-modal data elements from a variety of disparate sources (e.g., imaging, behavioral, or cognitive assessments), as well as descriptions of the derived results to ensure greater reproducibility and comparability of scientific findings across large-scale, heterogeneous, neuroimaging research projects.

A pilot project using the ARIES instantiation of PRISM is being conducted with collaborating investigative teams from the departments of Psychiatry, Neurology, and Biomedical Informatics at UAMS. This project aims to identify common pathways of neurodegeneration and candidate endophenotypes that could distinguish discrete pathophysiologic processes. This project includes neuroimaging measures (structural and functional MRI) as well as putative endophenotypic data obtained from a variety of assessment instruments designed to measure neuro-motor integration (e.g., wearable body sensors, gait-assessment floor mat, digitized gloves, speech samples, and handwriting/drawing assessments on a digitizing tablet), clinical disease characteristics (e.g., the Unified Parkinson's Disease Rating Scale) or neurocognitive functions (e.g., scores on standardized neuropsychological tests).

**Figure 1:**
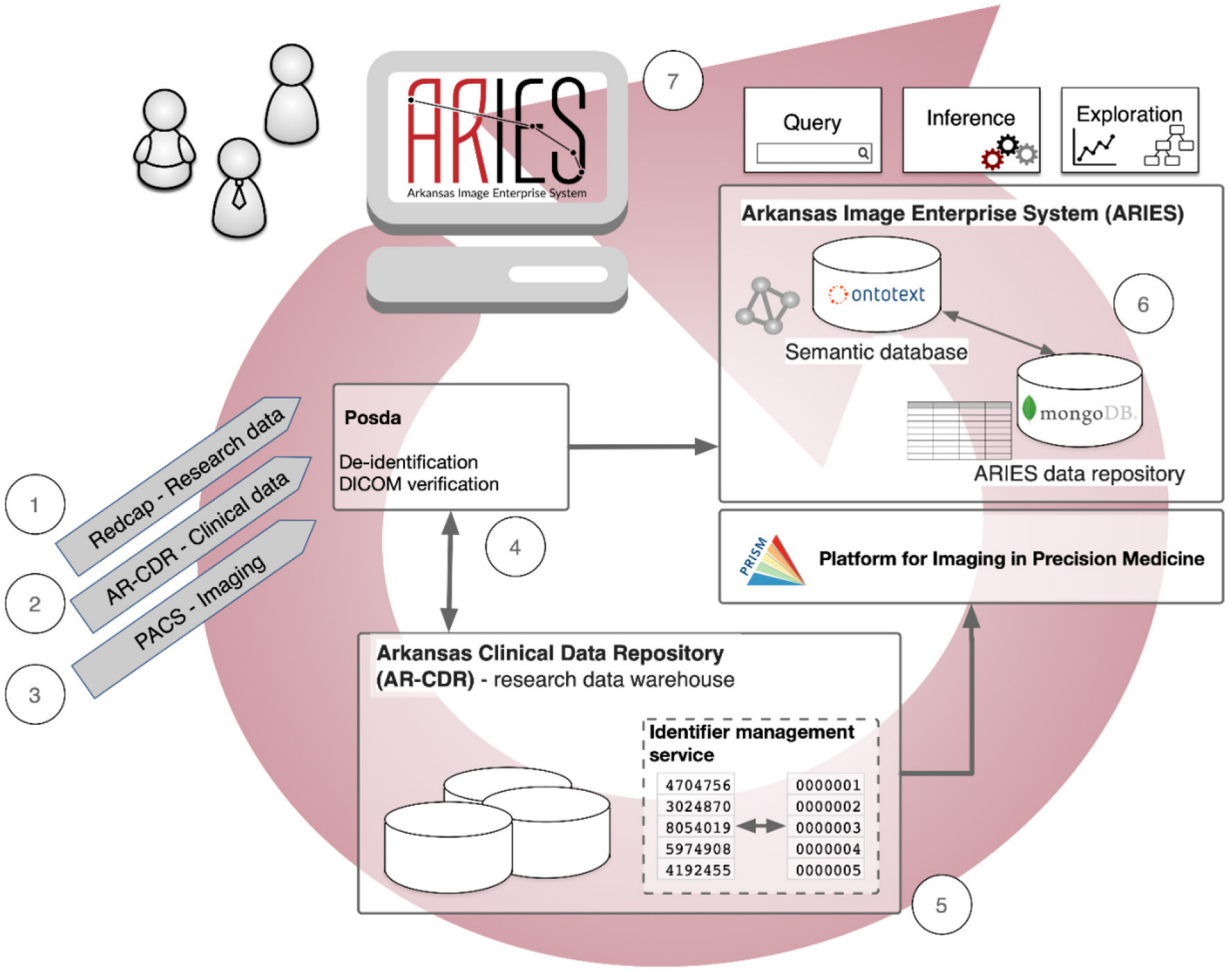
Data management pathways and informatics processes in the Arkansas Imaging Enterprise System (ARIES).

As shown in Figure 1, the data management pathways and informatics processes in our ongoing pilot project using ARIES are: (1) Research data collected from a dedicated electronic data capture application such as REDCap (or available as spreadsheets) are de-identified and transferred to a secure storage area for ingestion into ARIES. (2) Clinical and demographic information are retrieved from electronic medical records in the Arkansas Clinical Data Repository (AR-CDR) (Baghal et al., 2019) - using standard extract/transfer/load (ETL) operations. (3) Clinical and research imaging data are imported from the UAMS Picture Archiving and Communication System (PACS) using standard DICOM protocols. (4) These imaging data are deposited into the ARIES instance of the Perl Open Source DICOM Archive (POSDA) (Bennett et al., 2018), a data curation tool that tracks the time and date of image data receipt, manages unique data identifiers, and performs image verification and de-identification with change history. (5) Pseudonymous subject identifiers are generated to maintain secure linkages across the imaging and non-imaging data from AR-CDR using the On-Demand Cohort and API Subject Identifier Pseudonymization (O-CASP) (Syed et al., 2020) to ensure the anonymity of research subjects. (6) All de-identified data (research assessments, clinical information, demographics, and imaging) are collected into the ARIES data repository, which consists of a MongoDB NoSQL database (Banker, 2011; Han et al., 2011) as the default storage location for all non-image data, and a semantic database (triple-store) (Rohloff et al., 2007) to manage data integration and provide semantic representations of metadata and key components of study data. (7) Finally, the fully integrated data are then made available to tools for query, cohort specification, inferencing, and exploration.

This report presents PRISM's semantic integration approach and capabilities, as implemented in ARIES. Two unique study cohorts from this project were used, both containing data from individuals who had been diagnosed with Parkinson's disease (PD) and from healthy elderly individuals with no known diagnoses of a neurological or neurodegenerative condition. Among the research questions initially posed by the investigative teams was whether there were any significant differences in the volume of the hippocampus between individuals with or without a diagnosis of PD. A secondary question was whether any of the performance scores on the neuropsychological assessment instruments that were included were predictive of hippocampal volumes. This report details the semantic integration approach taken on this project, which is required to address research questions like this that combine elements from several disparate data types and sources. The general semantic integration approach in PRISM/ARIES is domain independent, suitable for a broad array of investigations using multi-modal imaging and non-imaging data.

## Methods

### Subjects

The datasets used in this study originate from two separate sources: Dataset1 includes neuroimaging (structural and resting-state functional MRI, as well as EEG), demographics, and assessment data collected on a longitudinal research study funded by the Michael J. Fox Foundation (MJFF). A total of 50 participants in this study for whom baseline structural MRI and neuropsychological assessment data were available were included in the current use-case analyses. Of these, 29 had been clinically diagnosed with idiopathic PD and 21 were elderly healthy control (HC) subjects. Dataset2 includes data compiled by the MJFF and made available to researchers as part of the Parkinson's Progression Markers Initiative (PPMI; http://www.ppmi-info.org/data) (Banker, 2011). For the current use-case analyses, baseline MRI, demographic, clinical, and neuropsychological assessment data were obtained from the PPMI repository for 100 individuals, of whom 69 had been clinically diagnosed with idiopathic PD and 31 were elderly HC subjects, with no known neurological diagnoses or first-degree relatives with PD. Both of the studies were conducted following review and approval from applicable Institutional Review Boards and all participants provided informed written consent that was fully compliant with the principles of the Declaration of Helsinki and included a clause stipulating that anonymized data could be used in secondary analyses such as the current project. Table 1 provides a summary of the demographic characteristics of participants.

**Table 1:** Demographic characteristics of study participants.

	**Dataset1**		**Dataset2**	
	**PD**	**HC**	**PD**	**HC**
Number of participants	29	21	69	31
% Female	28	62	34	39
Mean Age in Years	67.1	69.7	61.8	58.7
Mean Years of Education	17.9	17.1	16.4	17.2
Mean Hoehn and Yahr Score	2.29	0	1.65	0
Mean MoCA Total Score	25	26.6	26.9	28.1

### Neuropsychological Assessments

Each of the two datasets included in the current report employed batteries of standardized neuropsychological assessment instruments to characterize the cognitive functions of the participants. The battery administered to participants in Dataset1 included the Montreal Cognitive Assessment (MoCA) (Nasreddine et al., 2005), the Trailmaking Test Parts A and B (Greenlief et al., 1985), the Stroop Color-Word Interference Test (Stroop, 1935), and the Repeatable Battery for the Assessment of Neuropsychological Status (RBANS) (Randolph et al., 1998), which itself includes subtests for Verbal List Learning (with Delayed Recall and Recognition), Story Learning (and Delayed Recall), Figure Copy (and Delayed Recall), Line Orientation, Picture Naming, Digit Span, Coding, and Semantic/Category Fluency. The battery administered to participants in Dataset2 included the MoCA, the Semantic/Category Fluency Test (Rosen, 1980), the Phonetic/Letter Fluency Test (Benton et al., 1983) the Hopkins Verbal Learning Test – Revised (HVLT-R) (Brandt, 1991), the Letter-Number Sequencing Test (Gold et al., 1997), the Benton Judgement of Line Orientation Test (Benton et al., 1978), the Symbol-Digit Modalities Test (Smith, 1968), and the University of Pennsylvania Smell Identification Test (UPSIT) (Doty et al., 1984). Notably, there were only two assessment instruments that were administered on both datasets, the MoCA and the Semantic/Category Fluency Test (using “Animals” as the category). As such, these measures were prioritized for semantic integration on the current use-case analyses. The approach to semantic representation as described below could also be used to aggregate scores across matching functional domains, though, this would require additional transformations of the data outside the scope of the current demonstration.

### MRI Processing Pipeline

MRI images were acquired from different scanner devices using standard 3D T1-weighted acquisition sequences (e.g., MPRAGE or SPGR) for Dataset1, and the T1 images for Dataset2 were acquired on a Siemens 3T Trio scanner using a 3D MPRAGE acquisition sequence. All images were acquired with slice thickness of 1.5 mm or less with no interslice gaps. Processing of these T1 images was conducted using the following tools from the FMRIB Software Library (FSL) (Smith et al., 2004; Woolrich et al., 2009; Jenkinson et al., 2012): Brain extraction was performed using the Brain Extraction Tool (BET). Registration and subcortical segmentation was performed using the FMRIB's Integrated Registration and Segmentation Tool (FIRST) (Patenaude et al., 2011) which co-registers all images to a 1 x 1 x 1 mm resolution template from the Montreal Neurological Institute (MNI152) (Fonov et al., 2011) via 2-step linear registration using the FMRIB's Linear Image Registration Tool (FLIRT) (Jenkinson et al., 2002) before segmenting subcortical spaces into 15 regions that include the brainstem and seven bilateral structures (hippocampus, amygdala, nucleus accumbens, caudate, pallidum, putamen, and thalamus). Total intracranial volumes and hippocampal volumes were both calculated using the “fslstats” utility. Finally, hippocampal volumes were normalized for each participant by dividing by the total intracranial volume.

### Semantic Integration Approach

ARIES integrates and manages datasets and associated metadata using shared, ontology-based representations that account for both explicit and implicit connections among the data across the source datasets. This approach removes obstacles to: working with different source representations for the same type of information; connecting and interpreting different types of data that are about the same phenomena (e.g., different cognitive assessments that provide measurements of the same domains); and combining diverse data sets that are about the same individuals. ARIES semantically integrates diverse datasets using representations based on axiomatically rich ontologies to construct a knowledge graph that combines instance data from these unique study cohorts into a shared semantic representation. This knowledge graph is stored in a triple-store database that supports reasoning over and querying the integrated data. This approach facilitates discovery of important new linkages among endophenotypic expressions of disturbed neural functions and discrete neuroanatomical markers of neurodegeneration obtained from the derived neuroimaging results.

### Ontologies and Knowledge Graph

**Figure 2:**
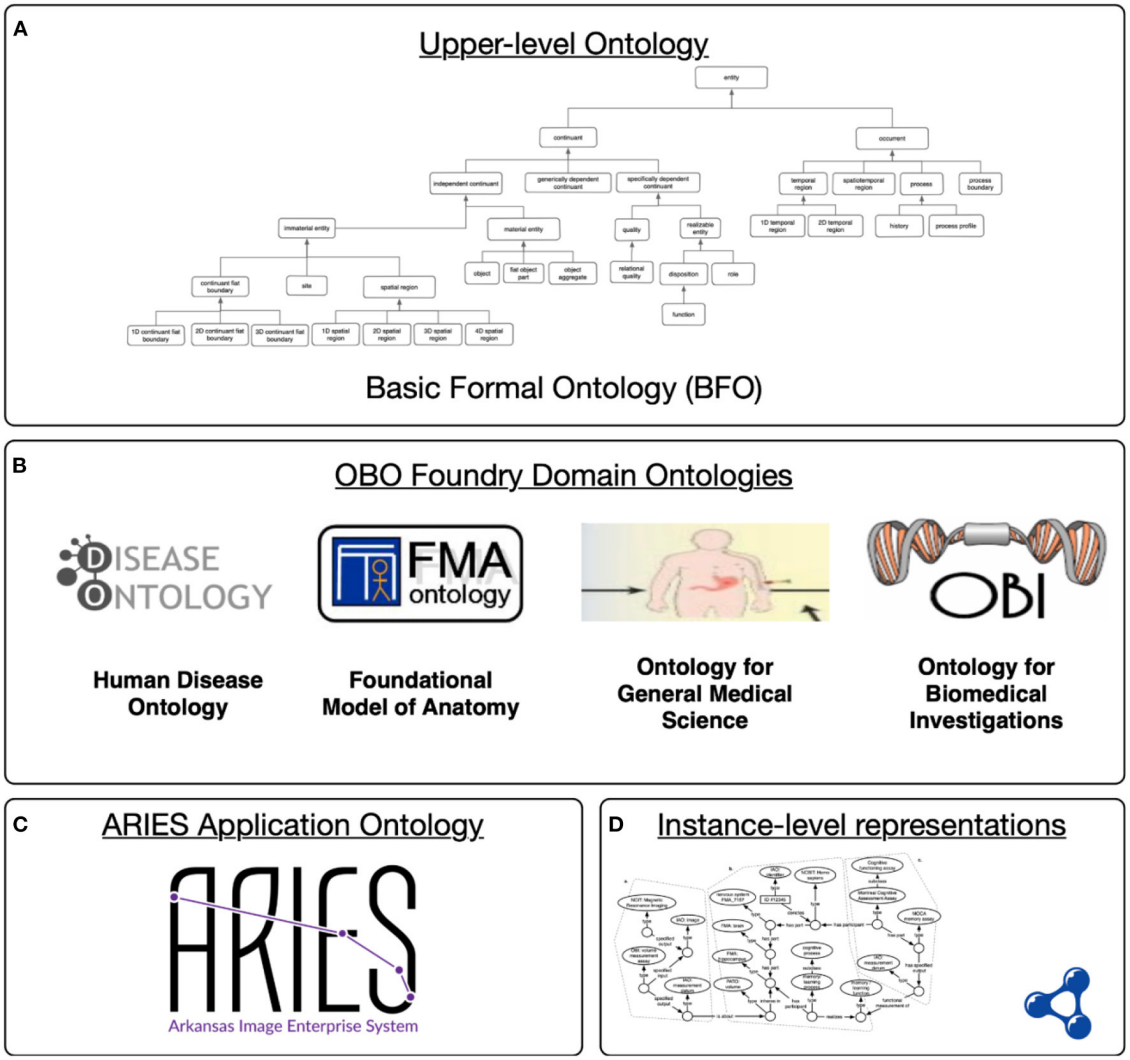
Levels in the ARIES semantic infrastructure: **(A)** The upper level ontology provides a common language; **(B)** domains ontology extend this to cover a particular area of science; **(C)** ARIES application-specific terms and definitions for our project; **(D)** ARIES knowledge graph containing instance data aligned with the domain and application ontologies.

Semantic representation of these data in ARIES relies on axiomatically rich ontologies from the Open Biomedical Ontologies (OBO) Foundry (Smith et al., 2007), which provides a collection of orthogonal and consistent biomedical ontologies aligned through their shared use of the *upper level* Basic Formal Ontology (BFO). As an upper level ontology, BFO provides a high-level model or theory of the types of entities that exist in the world (*processes, material entities*, and so on). Domain ontologies extend this upper level to model scientific knowledge in particular areas. As shown in Figure 2, the ARIES knowledge graph uses interconnected ontology resources at different levels of specificity, starting with the upper level BFO. A collection of interoperable domain ontologies is used to model foundational anatomy (FMA), biomedical investigations (OBI), and more. These ontologies provide background knowledge in a machine-interpretable, logical language (Smith et al., 2007; Blobel and Yang, 2018; Brochhausen et al., 2018). Of particular relevance is the Neuropsychological Testing Ontology (NPT) (Cox et al., 2013), an OBO-compatible ontology that provides detailed modelling of cognitive assessment plans and processes, of the measurements generated by those assessments, and of the underlying cognitive functions and processes they aim to measure.

All these ontologies are encoded using the Web Ontology Language (OWL) (Hitzler et al., 2009), a knowledge representation language based on description logics (DL) (Baader et al., 2003) and the Resource Description Framework (RDF) (McBride, 2004).

An *application ontology* is tailored to a specific application or use (Arp et al., 2015), commonly comprising data elements or entities specific to that application and that are unlikely to be used or developed further by a wider community. The ARIES application ontology developed as part of this project includes terms that are specifically useful for modelling our data, for instance the ARIES-application term “*right hippocampal volume feature extraction using the FIRST segmentation utility from FSL on a T1 MRI image”*. Such terms are given axiomatic definitions linking them to domain ontology terms such as OBI's “measurement datum” and the FMA term for “right hippocampus”. For example, the axiomatic definition of the term mentioned above includes, among others, assertions that:

- This specific type of feature extraction is a subtype of “*OBI*: *planned process”* and a subtype of “*ARIES*: *right hippocampal volume feature extraction”*.
- This type of process has as one of its specified inputs an “*IAO: image”*
- This type of process has as its specified output some “*ARIES*: *right hippocampal volume measurement datum”*.
- This type of process achieves a “*OBI: feature extraction objective”*

In the foregoing, “*OBI: planned process”* refers to the term labeled “planned process” in the OBI ontology, and similarly for terms from other ontologies. Within the actual axiomatic definitions of ARIES application ontology terms, each term from another ontology is included/referenced using its globally unique Internationalized Resource Identifier (IRI). For example, the IRI for “OBI: planned process” is http://purl.obolibrary.org/obo/OBI_0000011. OBI and related ontologies also include definitions for many of the standard relations used in their definitions, for example “*has specified input”* and “*achieves planned objective”*. These ontological resources are combined into a single knowledge graph within ARIES, linking the ontologies to instance-level representations of the entities that ARIES source data are about: subjects, their assessments, individual cognitive functions, brain regions, etc. This knowledge graph is stored in a triple-store database (“semantic database” in Figure 1) that supports querying and reasoning over the integrated data.

Though imaging workflows management within ARIES is still a work in progress, this capability will ensure reproducibility and provenance tracking by explicitly representing within the ARIES knowledge graph both the data processing pipelines themselves, and all derived results. The Neuroimaging Data Model (NIDM) is built on semantic web technologies and establishes vocabularies for neuroimaging experiments, workflows, and derived results (Keator et al., 2013). NIDM differs in its approach from the OBO Foundry resources we have deployed in ARIES, most notably in that it is not aligned with the BFO upper-level ontology, and so is not automatically interoperable with the rest of our knowledge graph. We are working to determine how NIDM can be combined with our knowledge graph to represent workflows and provenance.

## Results

### Mapping Instance Data

This pilot study combines data about subjects from two different studies, collected by different investigators at different times and in different places, and without any common representation scheme. While the semantic integration accomplished here goes beyond simple data harmonization, having these data harmonized into a common and clear representation is one immediate advantage. For example, both datasets include Montreal Cognitive Assessments (MoCA) taken by subjects, and both include scores for MoCA subtests, such as the MoCA language fluency / category fluency test. However, in one of our source data sets, this score is stored under the heading “*MOCA_words_total*”, while in the other it is stored more opaquely as “*MCAVF*”. That these fields capture the same type of information is not obvious to a human user of these data, who must understand the domain and consult any documentation accompanying the data to have any hopes of combining these. The background knowledge that a user of these data must either already have in order to use the data includes: that the MoCA language fluency test is a planned process; that it is part of the Montreal Cognitive Assessment; that its output is a measurement about a cognitive function of the subject; and so on. By integrating these instance data using ontologies that contain the relevant background information in both human- and machine-interpretable forms, we greatly enhance its potential for reuse, enable automated inference over these data using semantic web tools, and create a richly-labeled dataset amenable to use with other artificial intelligence approaches, including statistical and machine learning based uses of these data.

ARIES instance data are mapped into semantic representations using a semi-automated process that starts with construction of appropriate semantic patterns (subgraphs of ontology terms and instances) represented in the source data. These mappings are then processed, along with the source data, by a Python program to translate the source data into OWL/RDF-based representations, which are then serialized to RDF output files. Our Python program to accomplish the translation to RDF/OWL uses the open source RDFLib package (https://github.com/RDFLib/rdflib). The OBO-ROBOT (Jackson et al., 2019) command line tool is used to extract minimal modules from the relevant ontologies (OBI, NPT, etc.) that include all the terms used in our instance data, as well as recursively retrieving the terms that appear in their axioms. OBO-ROBOT is also used to merge ontology modules with our instance data and the ARIES application ontology. The resulting file is loaded into an Ontotext GraphDB triple-store (https://www.ontotext.com/products/graphdb/), which provides rulesets for various OWL/description logic reasoning profiles, as well as exploration and query APIs.

**Figure 3:**
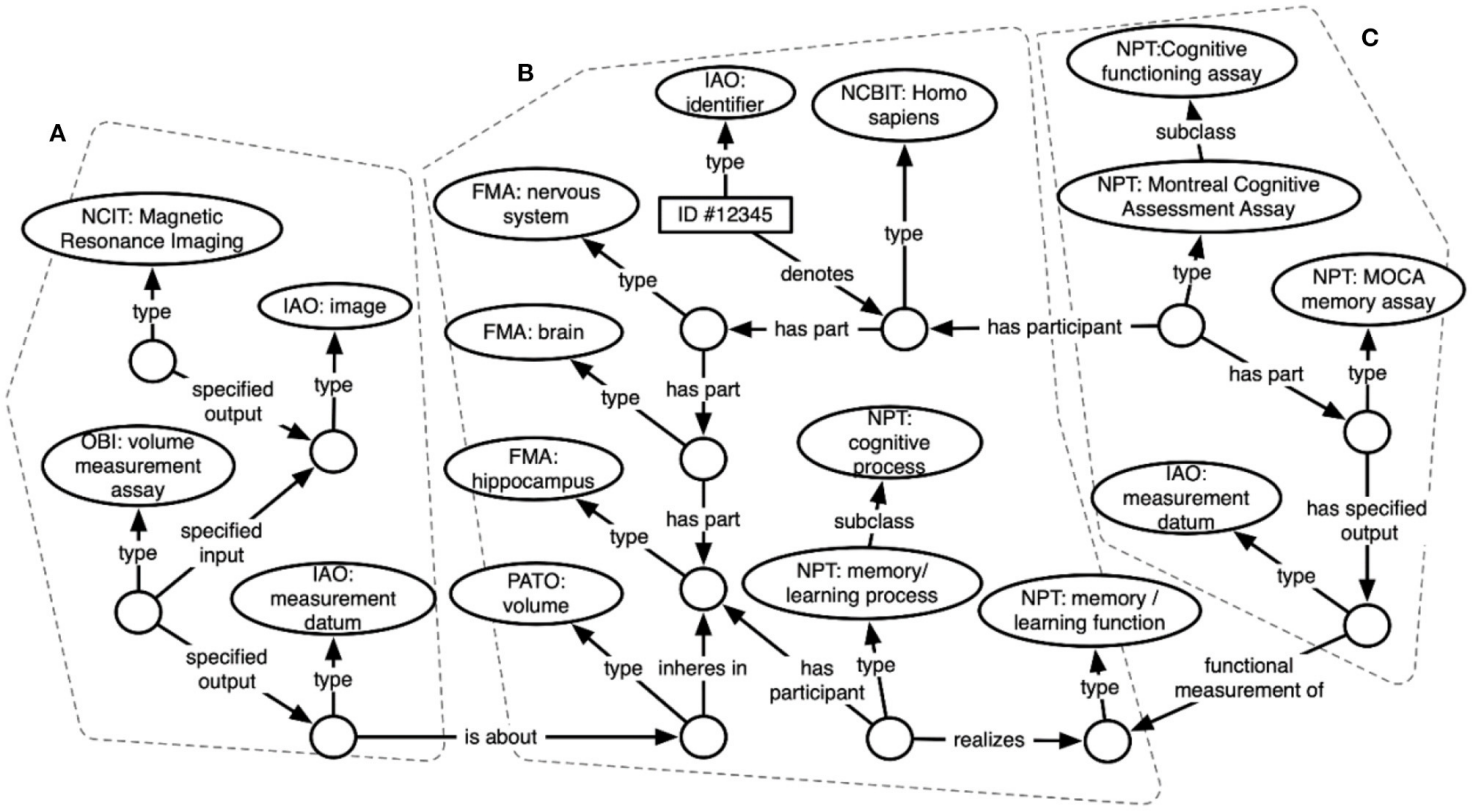
ARIES knowledge graph data structure showing ontology-based representations of **(A)** an image capture and image-derived volume measure, **(B)** a human subject, and **(C)** a cognitive assessment.

Figure 3 shows an excerpt of the resulting knowledge graph, focusing on representations for a subset of data about a single subject. Circles indicate RDF instances, while ovals indicate ontology classes. Arrows show relations between instances, or between instances and classes. In the center (**b**) is an instance of type “*Homo sapiens*” – this represents the study subject that these data are about. Attached to that instance is some of the knowledge contained in the FMA about human anatomy, including instances for the subject's brain and for parts of the subject's brain (hippocampus), as well as specific cognitive functions and processes realized within the brain. On the left side (**a**) are instances of brain imaging processes (MRI) and the image produced, as well as a volume measurement – an image-derived feature. These sections are linked by connecting the subject's hippocampus to the volume measurement that is about the hippocampus. The right side (**c**) shows representations of a cognitive assay that the subject has completed (an instance of the MoCA), and a specific part of that assay that measures memory function. These processes and the measurements they output are linked in the graph to the subject's memory function.

### Cohort Selection Using Semantically Integrated Data

Once compiled across the two datasets, the semantic representations of the data elements allow for the selection of cohorts for statistical comparisons and other analyses. Initially, we sought to select all subjects for whom hippocampal volumes were available. The PD subjects were also stratified according to total scores on the MoCA. The listing below shows a simple SPARQL Protocol and RDF Query Language (SPARQL) (Pérez et al., 2009) query used for cohort selection on data in the semantic repository. SPARQL queries use term IRIs to refer to ontology classes, relations, and individuals. For example, the IRI http://purl.obolibrary.org/obo/NPT_0020000 is the identifier for the “Montreal cognitive assessment assay” in the Neuropsychological Testing Ontology. This query uses SPARQL PREFIX statements to improve its readability for readers not familiar with the IRIs. SPARQL queries specify graph patterns with variables and return results that match those patterns. This query retrieves the subject identifier and total MoCA score for all subjects (across source data sets) that scored <26 on the MoCA. This query returns 110 subjects with total MoCA scores below 26, 78 from the MJFF source data set, and 32 from the PPMI data set. A more complex query could delve into the sub-scores (specific parts of the MoCA test), or even run across the knowledge graph to also retrieve image-derived features like volumetric measurements of specific brain regions, for instance, the hippocampus, as seen in Figure 2.

In addition to pattern-based querying with SPARQL, the triple-store also provides semantic web reasoning capabilities that can be used to further simplify retrieval and inference on relevant information from our repository. For example, we might define classes in the ARIES application ontology for “*Study subject with mild cognitive impairment*,” “*Study subject with reduced semantic category fluency function*,” or “*Study subject with reduced left hippocampal volume”* that incorporates into their axioms the necessary patterns for a reasoner to automatically group individual subjects into these classes based on the contents of the knowledge graph.

Work is ongoing in PRISM to develop ontology-driven tools for cohort selection, data exploration, and advanced search. These tools, which are being deployed and tested within ARIES, will allow researchers to take advantage of semantically integrated data in the knowledge graph without directly using SPARQL queries or other semantic web technologies.

Box 1 **A SPARQL query that retrieves the subject identifier and total MoCA score for all subjects across source data sets that scored less than 26 on the MoCA.**

PREFIX human: <http://purl.obolibrary.org/obo/NCBITaxon_9606>

PREFIX denotes: <http://purl.obolibrary.org/obo/IAO_0000219>

PREFIX has_participant: <http://purl.obolibrary.org/obo/BFO_0000057>

PREFIX moca: <http://purl.obolibrary.org/obo/NPT_0020000>

PREFIX assay: <http://purl.obolibrary.org/obo/OBI_0000070>

PREFIX has_specified_output: <http://purl.obolibrary.org/obo/OBI_0000299>

PREFIX value_specification: <http://purl.obolibrary.org/obo/OBI_0001933>

PREFIX measurement_datum: <http://purl.obolibrary.org/obo/IAO_0000109>

PREFIX has_value_specification: <http://purl.obolibrary.org/obo/OBI_0001938>

PREFIX has_specified_value: <http://purl.obolibrary.org/obo/OBI_0002135>

PREFIX subject_role: <http://purl.obolibrary.org/obo/OBI_0000097>

PREFIX inheres_in: <http://purl.obolibrary.org/obo/RO_0000052>

select ?idl ?score where {

?s rdf:type human:. # s is the individual subject; binds to a person

?srole rdf:type subject_role:. # ensures that the person is a subject

?srole inheres_in: ?s.

?id denotes: ?s. # the subject identifier

?id rdfs:label ?idl.

# the subject participated in a moca assay, scored <26 total

?assay has_participant: ?s.

?assay sesame:directType moca:.

?assay has_specified_output: ?output.

?output rdf:type measurement_datum:.

?output has_value_specification: ?vs.

?vs has_specified_value: ?score.

FILTER (?val < 26)

}

### Exploratory Analyses on Hippocampal Volumes

The initial question posed by the investigative team was whether there were any differences between PD and HC participants in hippocampal volumes. Normalized hippocampal volumes were compared between the PD and HC participants using a one-way analysis of variance. Although the mean normalized volume for PD participants was slightly smaller than those from HC participants, these differences were not found to be statistically significant (F = 2.72; *p* = 0.10). However, when the PD group was stratified into separate groups using the common MoCA threshold for cognitive impairment as those scoring <26, the normalized hippocampal volumes were found to be significantly different between the three groups (F = 3.67; *p* = 0.028). *Post-hoc* comparisons using Fisher's Least Significant Difference (LSD) method, revealed that the normalized hippocampal volumes for the PD patients who scored <26 on the MoCA were significantly smaller than those from PD patients who scored ≥26 (*p* = 0.039), as well as lower than the mean volumes for the HC group (*p* = 0.008).

These preliminary findings are consistent with prior research, which have shown increased hippocampal atrophy among PD patients with cognitive impairments or dementia (Laakso et al., 1996; Camicioli et al., 1999, 2003; Yildiz et al., 2015).

## Discussion

The Arkansas Imaging Enterprise System (ARIES) leverages the basic capabilities of the Platform for Imaging in Precision Medicine (PRISM) to effectively represent and integrate a diverse set of multi-modal data elements and provide detailed descriptions of the results obtained across the analytic processing stages and in relation to the supporting endophenotypic data. Such capabilities are essential to ensure greater reproducibility in large-scale neuroimaging research projects. In the current results, we have shown that the use of semantic representation patterns based on axiomatically rich ontologies facilitates combining, linking, and interpreting these data in a way that makes them more easily interpretable and reusable. The machine-interpretable description logic definitions accompanying the axiomatically rich ontologies used in our knowledge graph provide explicit semantics for the data necessary for machine reasoning to support inference, analysis, and exploration. Currently under development are ontology-aware user-facing tools backed by the knowledge graph that provide cohort selection and data exploration capabilities. The knowledge graph constitutes an integrated, richly-labeled dataset, a valuable feature-enriched resource useful for further analyses and approaches extending beyond knowledge representation and reasoning into other artificial intelligence approaches, including statistical and machine learning applications.

Future directions for the ongoing development of PRISM, as well as the ARIES instance, will also include semantic representations of the processing pipelines using tools such as the Neuroimaging Data Model (NIDM) (Keator et al., 2013) to describe the software applications, processing steps, and derived neuroimaging results. We also plan to integrate with the work being done by the ReproNim initiative (repronim.org) (Kennedy et al., 2019), which has developed a highly innovative suite of utilities and training modules to promote greater reproducibility in neuroimaging research. For example, ARIES is capable of storing containers within which executable processing pipelines will be housed. When used in conjunction with the ReproNim utilities, as well as the NIDM framework, the ARIES infrastructure offers a comprehensive solution for integrated semantic representation of all pertinent aspects of conducting a fully re-executable neuroimaging investigation such that researchers could share not only their findings, but also the detailed processing pipelines that would be required for other researchers to replicate their results. It is hoped that such functionality will help to alleviate the notoriously poor replicability of neuroimaging results that has plagued the field and limited the generalizability of many published discoveries (Kennedy et al., 2019).

## Data Availability Statement

The original contributions presented in the study are included in the article/supplementary material, further inquiries can be directed to the corresponding author/s.

## Author Contributions

JB leads semantic integration efforts in PRISM and in the ARIES instance detailed in this manuscript. AK developed the processing pipelines and conducted statistical analyses on the imaging data. AD implemented the processing pipeline to derive hippocampal and intracranial volumes on Dataset1, while CC implemented these processing steps on Dataset2. JB and AK served as the primary authors of the manuscript. All other authors contributed to the formulation, revision, and editing of the resulting publication and also contributed to the development and execution of the studies from which the ARIES pilot project described herein derived its source data.

## Funding

This project has been funded in whole or in part with federal funds from the National Cancer Institute, National Institutes of Health under Contract No. HHSN261200800001E. The content of this publication does not necessarily reflect the views or policies of the Department of Health and Human Services, nor does mention of trade names, commercial products, or organizations imply endorsement by the U.S. Government. Under this contract the University of Arkansas is funded by Leidos Biomedical Research subcontract 16X011. Funding was also provided by U24CA215109. This project is supported in part by the UAMS Translational Research Institute (TRI), grant UL1 TR003107 through the National Center for Advancing Translational Sciences of the National Institutes of Health (NIH). The content is solely the responsibility of the authors and does not necessarily represent the official views of the NIH. Additional support was provided by a competitive research grant (PI: Galvin) from the Michael J. Fox Foundation for Parkinson's Research, which had no direct involvement in the collection, analysis, or interpretation of the data presented herein. Data used in the preparation of this article were obtained from the Parkinson's Progression Markers Initiative (PPMI) database (www.ppmi-info.org/data). PPMI is a public-private partnership funded by the Michael J. Fox Foundation for Parkinson's Research and funding partners [a listing of all of the PPMI funding partners can be found at www.ppmi-info.org/fundingpartners].

## Conflict of Interest

The authors declare that the research was conducted in the absence of any commercial or financial relationships that could be construed as a potential conflict of interest.

## Publisher's Note

All claims expressed in this article are solely those of the authors and do not necessarily represent those of their affiliated organizations, or those of the publisher, the editors and the reviewers. Any product that may be evaluated in this article, or claim that may be made by its manufacturer, is not guaranteed or endorsed by the publisher.

## Abbreviations

ADNI: Alzheimer's Disease Neuroimaging Initiative
AR-CDR: Arkansas Clinical Data Repository
ARIES: The Arkansas Imaging Enterprise System
BET: Brain Extraction Tool
BFO: Basic Formal Ontology
DICOM: Digital Imaging and Communications in Medicine
DL: description logics
EEG: electroencephalography
ETL: extract/transfer/load
FLIRT: FMRIB's Linear Image Registration Tool
FMA: Foundational Model of Anatomy
HC: healthy control
HVLT-R: Hopkins Verbal Learning Test-Revised
LSD: Fisher's Least Significant Difference
MEG: magnetoencephalography
MJFF: Michael J. Fox Foundation
MNI152: Montreal Neurological Institute 1 x 1 x 1 mm resolution template
MoCA: Montreal Cognitive Assessment
MPRAGE: Magnetization Prepared–RApid Gradient Echo
MRI: magnetic resonance imaging
NIDM: Neuroimaging Data Model
NPT: Neuropsychological Testing Ontology
O-CASP: On-Demand Cohort and API Subject Identifier Pseudonymization
OBI: Ontology for Biomedical Investigations
OBO: Open Biomedical Ontologies
OWL: Web Ontology Language
PACS: Picture Archiving and Communication System
PD: Parkinson's disease
PET: positron emission tomography
POSDA: Perl Open Source DICOM Archive
PPMI: Progressive Parkinson's Markers Initiative
PRISM: Platform for Imaging in Precision Medicine
RBANS: Repeatable Battery for the Assessment of Neuropsychological Status
RDF: Resource Description Framework
SPARQL: SPARQL Protocol and RDF Query Language
SPGR: Spoiled gradient recalled echo
TCIA: The Cancer Imaging Archive
UAMS: University of Arkansas for Medical Sciences
UPSIT: University of Pennsylvania Smell Identification Test.
